# Towards an odour-baited trap to control *Musca sorbens*, the putative vector of trachoma

**DOI:** 10.1038/s41598-021-91609-1

**Published:** 2021-07-09

**Authors:** Ailie Robinson, Jack Bickford-Smith, Oumer Abdurahman Shafi, Muluadam Abraham Aga, Gemeda Shuka, Dereje Debela, Gebreyes Hordofa, Wondu Alemayehu, Virginia Sarah, Anna Last, David MacLeod, Matthew J. Burton, James G. Logan

**Affiliations:** 1 Department of Disease Control, London School of Hygiene and Tropical Medicine, Keppel Street, London, WC1E 7HT UK; 2 The Fred Hollows Foundation, P.O. Box 6307, Addis Ababa, Ethiopia; 3 Department of Clinical Research, London School of Hygiene and Tropical Medicine, Keppel Street, London, WC1E 7HT UK; 4 Global Partnerships Executive, The Fred Hollows Foundation, 12-15 Crawford Mews, York Street, London, W1H1LX UK; 5 Department of Infectious Disease Epidemiology, London School of Hygiene and Tropical Medicine, Keppel Street, London, WC1E 7HT UK; 6 Present Address: Population Service International, Addis Ababa, Ethiopia; 7 Present Address: Netherlands Development Organization, The Hague, The Netherlands

**Keywords:** Bacterial infection, Entomology

## Abstract

*Musca sorbens* is a synanthropic filth fly that aggressively attacks people to feed from mucous membranes of the eyes, nose or mouth, from open sores, or from sweat. It has long been suspected that this fly contributes to the transmission of eye infections, particularly trachoma, and recent work has added to the evidence base that *M. sorbens* is a trachoma vector in Ethiopia. There are few options to control *M. sorbens*, largely due to a lack of evidence. Space spraying with insecticides is effective, but an environmentally sound and long-term sustainable solution would be better, for example, mass trapping. We tested commercially available and homemade trap types in a pilot (laboratory) study and three field studies. A homemade design, built from a bucket and two empty water bottles, baited with a commercially available lure, The Buzz, was found to be most effective. This trap caught 3848* M. sorbens* over 26 trap ‘events’ (3- or 4-day periods); mean/median per 24 h 43.6 (standard deviation 137.10)/2.25 (IQR 0.25–12.67). The Buzz lure is cheap and effective for 4 weeks, and trap components cheap and locally available. Further studies are needed to understand the impact of this trap on local fly populations and the local transmission of trachoma.

## Introduction

*Musca sorbens*, the Bazaar fly, is thought to contribute to transmission of the blinding eye disease trachoma in certain regions of the world^1–5^. This species of ‘filth fly’ persistently attacks people, feeding on sweat, mucous secretions (particularly ocular and nasal), and any type of skin lesions including wounds, ulcers and even tiny breaks in the skin^1,6^. As the bacterium that causes trachoma, *Chlamydia trachomatis* (*Ct*), is found in ocular and nasal secretions, it is presumed that *M. sorbens* mechanically transmits this pathogen between people on, or in, it’s body^6^. Trachoma is a neglected tropical disease. It is estimated that 136.9 million people live in trachoma-endemic areas and 2 million are living with trachomatis trichiasis, where the disease has progressed to inturned eyelashes^7^. The contribution of eye-seeking flies to trachoma transmission remains poorly defined and understood, and more studies are needed to clarify their role as vectors. A cluster-randomised controlled trial (RCT) of fly control alone in The Gambia reduced trachoma prevalence^5^, and a cluster-RCT of fly control combined with antibiotic treatment in Tanzania found some evidence that fly control reduced trachoma prevalence at 6 months^8^. Additionally, as this species breeds in faeces, favouring human^1,9^, there is the potential for mechanical transmission of enteric pathogens^10–12^. A possible role in the transmission of Yaws (*T. pallidum* subsp. *pertenue*^13–15^) and the blood parasites *Trypanosoma brucei* and *Leishmania* spp.^16^ has been suggested. Aside from probable transmission of pathogens, the aggressive face-seeking behaviour of *M. sorbens* causes people, particularly children, considerable aggravation across its geographical range (much of the tropics and sub-tropics, excluding the new world).

Previous work explored the very strong attraction of *M. sorbens* to the odour of human faeces, identifying specific chemical components as probable oviposition cues^17^. While these compounds, including carboxylic acids, cresols and indole, are candidate *M. sorbens*-specific attractants^17^, further research investment is needed to refine a field-ready lure. Although a significant nuisance species and probable disease vector, scant options for controlling *M. sorbens* are available. Insecticide spraying with pyrethroids has been successful^5^, and treating human faeces with hexachlorocyclohexane resulted in a reduction in *M. sorbens*^18^. However, the development of resistance is known to be rapid in Muscid species and housefly resistance to pyrethroids is widespread globally^19^. Environmentally friendly alternatives to insecticide spraying are increasingly sought after. As with all filth flies, the most effective and desirable method of population control is total removal of the breeding site and larval food via sanitary advances (i.e. ‘preventative measures’). However, trachoma is found in resource-poor settings, often affecting the world’s poorest populations, where sanitation and hygiene are inaccessible. Further to that, *M. sorbens* has been documented to breed in the faeces of domestic livestock^1^, so improving human sanitation may not necessarily eliminate breeding. Fly trapping may be a cost-effective tool for *M. sorbens* control, and a desirable alternative to insecticide. Some trapping methods have been used to monitor *M. sorbens* populations, including WHO exit traps attached to plastic buckets and baited with fish^2^ or beef^20^, sticky traps (sheeting of some type coated with adhesive but not baited)^2,8,12^, and sticky pots (bait-containing pots with sticky lids, ‘Bristow traps’)^12,17^.

Mass trapping for population control uses chemical attractants to lure insects to a trap where they are confined and die^21^. Because of the importance of filth flies to the livestock industry, mass trapping of these pest species has been used in some high-income agricultural settings. In a study conducted on a dairy farm, more than 80,000 *M. domestica* were caught in 7 out of 9 weeks (only trapping 4 days per week) using ‘Spider Web’ traps (Sticky paper traps)^22^, although fly density at this type of location would have probably been very high. Other studies have suggested that mass trapping would be a feasible means to control blowflies^23^. As well as the agricultural industry, there is a significant domestic market in high-income countries for filth fly control tools that can be deployed in and around people’s homes. This has led to the development of many commercially available fly traps of variable efficacy^23–25^. Many of these use dehydrated food substances: broad-range attractants that can lure a wide range of filth fly species. In the present study, we hypothesised that these simple lures might be effective on *M. sorbens*, which is known to feed on rotting foods^6^, and shortcut the development of a potentially complex, species-specific, synthetic chemical lure.

In regions where filth flies may play an important role in the transmission of disease, the nuisance caused by high fly densities may contribute to good uptake and community engagement with fly traps for control around the household. This may particularly apply to the face-seeking *M. sorbens*. A cheap, easily deployable and non-noxious/non-toxic fly trap may constitute a tool that empowers individuals living in trachoma-endemic regions to reduce their household’s exposure to trachoma and other fly-borne pathogens, while improving hygiene around the home. The purpose of this study was therefore to couple a pre-existing, commercially available, broad-range lure with a contextually appropriate trap design for *M. sorbens*, by testing a range of both homemade and commercially available trap designs and lures.

## Results

### Pilot

**Figure 1:**
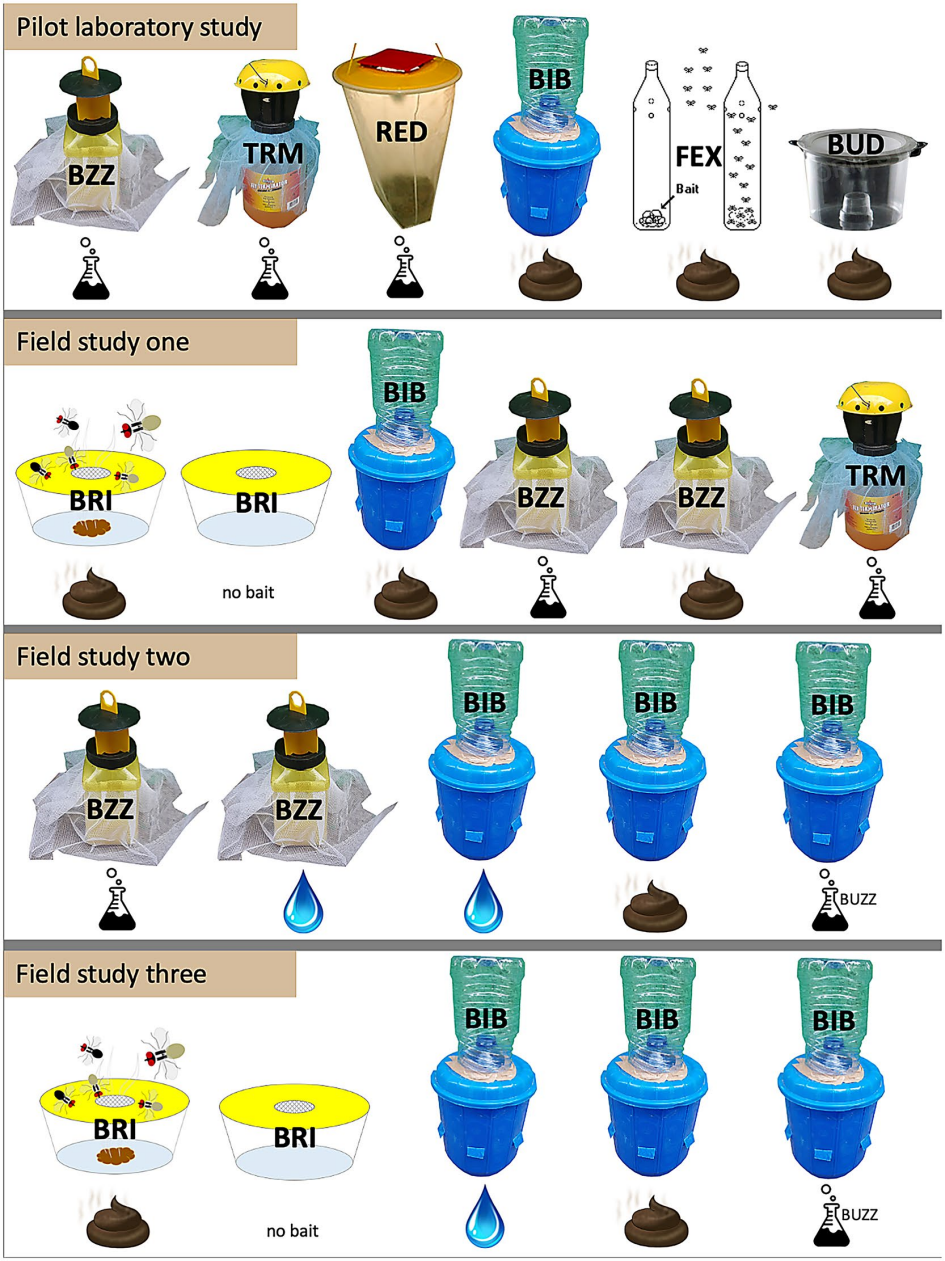
Trap types (design/bait combinations) used in the study. Trap designs are, row one (left to right): The Buzz (BZZ), Terminator (TRM), Red Top (RED), Bickford Bucket (BIB), Field Expedient (FEX), BugDorm (BUD). Row two additionally shows the Bristow trap (BRI, first/second left). Bait types used are shown under each trap design: Erlenmeyer flask (commercial lure of above trap), faeces cartoon (faeces lure), droplet (water lure). Erlenmeyer (BUZZ) indicates The Buzz lure was used with the above trap.

In a laboratory-based pilot study using colonised *M. sorbens,* a selection of commercially available and homemade trap types was tested (Fig. 1). The pilot study indicated that The Buzz may be an effective trap for catching *M. sorbens* (48.1% flies caught) (Table 1). The next most effective was the Terminator (36.4%), while the homemade Bickford Bucket (BB) trap was the most effective homemade design (23.5%) and the third most effective trap overall. The Field Expedient and Red Top trap caught very low numbers (10.8% and 4.3% flies respectively), the BugDorm caught no flies. As well as moderate success in catching flies, The Buzz, the BB and the Terminator were found to be easy to use and considered potentially appropriate to the field setting.

**Table 1:** Total *M. sorbens* caught by pilot laboratory testing of possible traps.

	Total flies released	F	M	Total caught (%)	F caught (%)	M caught (%)
Terminator	107	74	33	39 (36.4%)	26 (35.1%)	13 (39.4%)
The Buzz	106	73	33	51 (48.1%)	36 (49.3%)	15 (45.5%)
Red Top	93	62	31	4 (4.3%)	3 (4.8%)	1 (3.2%)
BugDorm	99	66	33	0 (0%)	0 (0%)	0 (0%)
Field expedient	93	53	40	10 (10.8%)	6 (11.3%)	4 (10.0%)
Bickford Bucket	98	69	28	23 (23.5%)	17 (24.6%)	6 (21.4%)

The number of flies introduced into the testing cage varied slightly due to deaths during acclimatisation.

*F* females, *M* males.

### Field study 1

**Figure 2:**
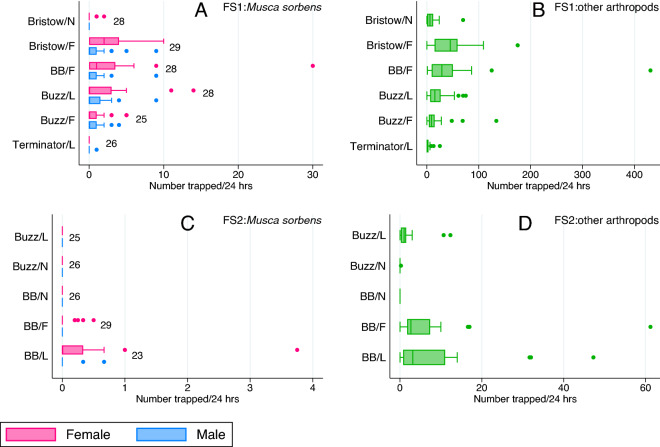
Male and female *M. sorbens* (**A**/**C**), and total ‘other arthropods’ (**B**/**D**) caught by different trap designs in field studies one (FS1) and two (FS2) (boxes, median and interquartile range; points, outliers). Trap abbreviations are: *Bristow/N* Bristow trap/no bait, *Bristow/F* Bristow trap/faeces bait, *BB/F* Bickford bucket/faeces bait, *Buzz/L* Buzz trap/Buzz lure, *Buzz/F* Buzz trap/faeces bait, *Terminator* Terminator trap/terminator lure, *Buzz/N* Buzz trap/no lure (water), *BB/N* Bickford bucket/no lure (water), *BB/L* Bickford bucket/lure (Buzz). Trap events per trap type given as data labels in (**A**) and (**C**); those are equal for (**A**/**B**) and (**C**/**D**). In FS2 trap events were approximately 72 h; data shown is per 24-h period.

In our first field study, a total of 325 *M. sorbens* were caught by all trap types (25–29 trap days per type; Table 2, Fig. 2A); of these 224 were female (68.9%) and 101 were male (31.1%). Greater numbers of non-*M. sorbens* arthropods were caught, with a total of 4070 caught by all trap types together (Table 3, Fig. 2B). Trap catch (female/male/total *M. sorbens* and non-*M. sorbens*) was strongly affected by the type of trap used (*P* < 0.001, Tables 2, 3). Although household and day affected *M. sorbens* trap catch, no measured household or environmental characteristic explained these effects. There was some evidence that the presence of rain (measured at trap collection) reduced the non-*M. sorbens* trap catch (*P* = 0.003; Table 3). The ‘positive control’ (Bristow trap baited with faeces) caught the greatest total *M. sorbens* and total other arthropods, catching 21.8 and 4.9 times more than the negative control respectively (rate ratio [RR]; 95% confidence intervals [CI] 6.72–70.45; 3.08–7.66; both *P* < 0.001). Interestingly, the homemade BB trap (faeces-baited) caught 17.5 times more female *M. sorbens* than the negative control (RR; 95% CI 5.47–55.85, *P* < 0.001), therefore performing better than the positive control that caught 15.0 times more than the negative control (RR; 95% CI 4.67–47.96, *P* < 0.001). The commercial Buzz trap caught 6.7 times more female *M. sorbens* than the negative control (RR; 95% CI 1.95–23.23, *P* = 0.003), which led to the idea of adding The Buzz commercial lure to the BB trap design in field study 2. In the positive control, we found 64 female and 31 male *M. sorbens*, and there was no evidence that there was a difference in sex ratio in the other three traps that caught *M. sorbens* (Buzz/L, Buzz/F and BB/F; *P* = 0.87).

**Table 2:** Female and total *M. sorbens* caught by different trap types across field studies one and three.

	*M. sorbens* (females)			*M. sorbens* (total)		
	Rate ratio (95% CI)	*P* value^(A)^	*P* value^(B)^	Rate ratio (95% CI)	*P* value^(A)^	*P* value^(B)^
Field study 1						
Bristow/N	Baseline^(C)^			Baseline^(C)^		
Bristow/F	14.97 (4.67–47.96)	< 0.001	< 0.001	21.76 (6.72–70.45)	< 0.001	< 0.001
BB/F	17.48 (5.47–55.85)	< 0.001		21.57 (6.60–70.47)	< 0.001	
Buzz/L	6.73 (1.95–23.23)	0.003		10.39 (3.07–35.20)	< 0.001	
Buzz/F	11.24 (3.46–36.47)	< 0.001		15.05 (4.56–49.67)	< 0.001	
Terminator/L	0 (0–0)	0.999		0.32 (0.03–3.03)	0.317	
Field study 3						
Bristow/N	Baseline			Baseline		
Bristow/F	8.70 (2.96–25.55)	< 0.001	< 0.001	7.29 (2.74–19.41)	< 0.001	< 0.001
BB/N	0 (0–0)	0.996		0.29 (0.03–2.48)	0.257	
BB/F	3.28 (1.03–10.44)	0.044		2.66 (0.92–7.75)	0.072	
BB/L	10.53 (3.55–31.22)	< 0.001		8.42 (3.13–22.67)	< 0.001	

Trap types are: Bristow trap/negative (Bristow/N; no bait), Bristow/faeces (Bristow/F), Bickford Bucket/faeces (BB/F), The Buzz/The Buzz lure (Buzz/L), The Buzz/faeces (Buzz/F), Terminator/Terminator lure (Terminator/L), Bickford Bucket/negative (BB/N) Bickford Bucket/ The Buzz lure (BB/L). Too few *M. sorbens* were caught in field study 2 to warrant statistical analysis.

^(A)^*P*-value comparing this category with baseline.

^(B)^*P*-value testing hypothesis that trap type is associated with number of flies trapped.

^(C)^Analysis adjusted for Day.

**Table 3:** Non-*M. sorbens* arthropods caught by different trap types across three field studies.

	Non-*M. sorbens* arthropods (total)		
	Rate ratio (95% CI)	*P* value^(A)^	*P* value^(B)^
Field study 1			
Bristow/N	Baseline^(C)^		
Bristow/F	4.86 (3.08–7.66)	< 0.001	< 0.001
BB/F	4.44 (2.79–7.06)	< 0.001	
Buzz/L	1.69 (1.00–2.85)	0.050	
Buzz/F	2.31 (1.41–3.79)	0.001	
Terminator/L	0.41 (0.21–0.79)	0.008	
Rain during trap collection (baseline no)	0.43 (0.23–0.81)	0.009	0.003
Field study 2			
Buzz/L	Baseline^(C)^		
Buzz/N	0.04 (0.01–0.31)	0.002	< 0.001
BB/N	0.00 (0-.)^(D)^	0.999	
BB/F	2.45 (1.39–4.29)	0.002	
BB/L	3.92 (2.19–7.02)	< 0.001	
Field study 3			
Bristow/N	Baseline		
Bristow/F	4.40 (1.96–9.89)	< 0.001	< 0.001
BB/N	0 (0–0)	0.998	
BB/F	1.66 (0.66–4.18)	0.278	
BB/L	5.59 (2.44–12.80)	< 0.001	
Rain since previous visit (baseline no)	0.44 (0.26–0.77)	0.004	0.003
Human faeces in compound (baseline none)	2.05 (1.01–4.16)	0.047	0.067
Sheep in compound	0.16 (0.03–0.87)	0.034	0.029

Trap types are: Bristow trap/negative (Bristow/N; no bait), Bristow/faeces (Bristow/F), Bickford Bucket/faeces (BB/F), The Buzz/The Buzz lure (Buzz/L), The Buzz/faeces (Buzz/F), Buzz/negative (Buzz/N [water]), Terminator/Terminator lure (Terminator/L), Bickford Bucket/negative (BB/N) Bickford Bucket/ The Buzz lure (BB/L).

^(A)^*P*-value comparing this category with baseline.

^(B)^*P*-value testing hypothesis that trap type is associated with number of flies trapped.

^(C)^Analysis adjusted for Day.

^(D)^Value too small to be calculated.

### Field study 2

In field study 2, a total of 34 *M. sorbens* were caught by all trap types across all 23–29 trap nights. The BB/lure trap was responsible for most of these captures (catching 24 female and three male *M. sorbens*), followed by the BB/faeces (seven female *M. sorbens*) then The Buzz/commercial trap (one female *M. sorbens*. No other traps caught *M. sorbens* in this study (Fig. 2C), statistical analysis of this low *M. sorbens* catch was not, therefore, appropriate. A total of 1842 non-*M. sorbens* arthropods were caught by all trap types together across trap nights (Fig. 2D). Relative to the commercial Buzz trap, the BB/faeces caught 2.45 times more non-*M. sorbens* arthropods (RR; 95% CI 1.39–4.29, *P* = 0.002; Table 3), however, the BB/Buzz lure combination caught 3.92 times more (RR; 95% CI 2.19–7.02, *P* < 0.001; Table 3).

### Field study 3

**Figure 3:**
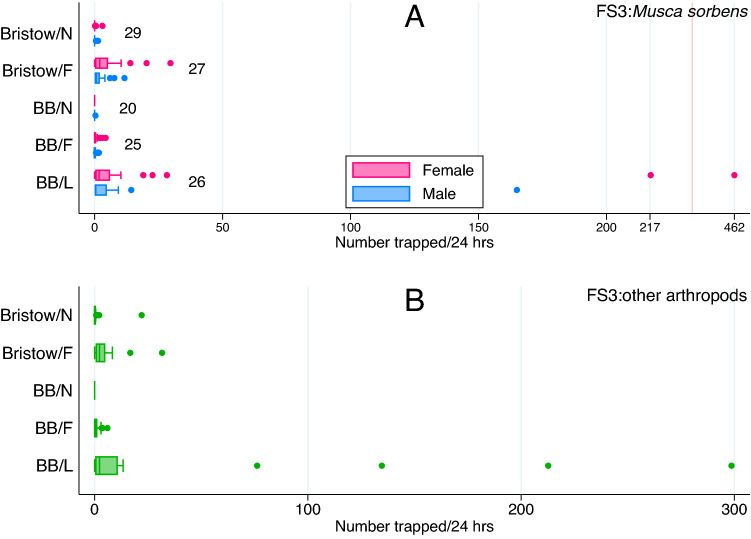
Male and female *M. sorbens* (**A**), and total ‘other arthropods’ (**B**) caught by different trap designs in field study 3 (FS3) (boxes, median and interquartile range; points, outliers). Trap abbreviations are: *Bristow/N* Bristow trap/no bait, *Bristow/F* Bristow trap/faeces bait, *BB/N* Bickford bucket/no lure (water), *BB/F* Bickford bucket/faeces bait, *BB/L* Bickford bucket/lure (Buzz). Trap events (3- or 4-day deployment) per trap type given as data labels in (**A**); those are equal for (**A**/**B**).

In field study 3, a total of 4510 *M. sorbens* were caught by all trap types across all 20–29 trap nights (Fig. 3A); of these, 3098 (68.69%) were female. A total of 3720 non-*M. sorbens* arthropods were caught across all trap types (Fig. 3B). The most successful trap type was the BB baited with the commercial Buzz lure, which caught 3848 *M. sorbens* (over 26 trap events, Supplementary Table S2), mean/median per 24 h 43.6/2.25 (standard deviation 137.10/interquartile range [IQR] 0.25–12.67; 3- or 4-day trap catches adjusted to represent 24-h catches).

There was a strong association between trap type and number of female, male and total *M. sorbens* (Table 2), and total ‘other arthropods’ (Table 3). Relative to the negative control (Bristow trap; neg [no bait]), the BB/buzzlure caught 10.53 times and the BB/faeces caught 3.28 times more female *M. sorbens* (RR; 95% CI 3.55–31.22, *P* < 0.001; 95% CI 1.03–10.44, *P* = 0.044 respectively; Table 2). The BB/buzzlure caught 8.39 times more male *M. sorbens* and 8.42 times more total *M. sorbens* than the negative control (RR; 95% CI 2.39–29.37, *P* = 0.001; 95% CI 3.13–22.67, *P* < 0.001 respectively; Table 2).

There was weak evidence that the household having a latrine positively affected the number of male *M. sorbens* trapped (RR 2.07; *P* = 0.089). While individual households (trapsites) caught different numbers of *M. sorbens*, no measured household characteristic explained these differences. The BB/buzzlure traps caught an exceptional number of *M. sorbens* on two occasions: 1399 and 1881 total *M. sorbens* were collected on 11/02/2019 at household 8 and on 14/02/2019 at household 10, respectively (349.75 and 627 per 24 h) (Supplementary Fig. S3). However, after omitting data from those collections a large effect of the BB/buzzlure remained (female *M. sorbens* RR 9.26, 95% CI 2.67–32.11, *P* < 0.001; male *M. sorbens* RR 5.49, 95% CI 1.53–19.74, *P* = 0.009; total *M. sorbens* RR 7.09, 95% CI 2.36–21.32, *P* < 0.001). The BB/buzzlure caught 5.59 times more non-*M. sorbens* arthropods than the negative control (RR; 95% CI 2.44–12.80, *P* < 0.001; Table 3), and there was some evidence that this trap catch was lower after rainfall (RR 0.44, *P* = 0.003; Table 3) and higher if human faeces were observed in the compound (RR 2.05, *P* = 0.067; Table 3). The presence of sheep was also found to be protective, with fewer non-*M. sorbens* arthropods caught when sheep were observed in the compound (RR = 0.16, *P* = 0.029; Table 3). In the positive control (Bristow trap; human faeces bait), a total of 388 (69.03%) female and 152 (27.05%) male *M. sorbens* were caught over 27 trap events; we found no evidence that there was a difference in sex ratio in the other traps that caught *M. sorbens* (BB/faeces, BB/buzzlure, Bristow/neg; *P* = 0.98).

## Discussion

A range of fly traps were tested for their efficacy in catching *M. sorbens*, and other arthropods, around households in Oromia, Ethiopia. Both commercially available traps (domestic market) and some homemade designs were trialled; homemade traps were baited with either human faeces (highly attractive to coprophagic *M. sorbens*^1,9^) or an attractant from one of the commercial traps. Over three field studies, iterative testing revealed the most effective and appropriate trap for this setting, a homemade trap design, the ‘Bickford Bucket’, baited with lure from the commercial trap The Buzz. In the final field study, conducted in the hot/dry season when *M. sorbens* are most prevalent, this trap/lure combination was successful in catching both *M. sorbens* and other arthropods.

The commercial traps The Buzz and the Terminator were tested in field study 1. The Terminator was not found to be useful here in trapping *M. sorbens* or other arthropods, and while The Buzz was relatively successful in field study 1, the trap design was not appropriate to the setting, being too easily saturated. Field studies 1 and 2 were conducted outside of the hot season when flies are less prevalent. For this reason, trap catches were overall lower, however, we were still able to compare trap type performance within each individual study. In field study 3, The Buzz lure was appropriated to the most useful and successful homemade trap design, the Bickford Bucket, and tested against a *M. sorbens* monitoring trap as a positive control (the Bristow Trap). The Bristow trap is baited with a sample of human faeces and relies on sticky paper to catch flies. This is not an optimal design because of the use of human faeces, and because sticky paper saturates and needs replaced. In rural poor locations where trachoma and *M. sorbens* are endemic, the more trap components that need replaced, the more logistically and financially problematic a trapping programme becomes. The Buzz lure is a non-toxic, protein-based product that is safe for deployment around the household.

The Bickford Bucket/Buzz lure combination was found to be the most successful trap for catching *M. sorbens*, in field study 3 this trap caught a total of 3848 *M. sorbens* (over 26 trap events), mean/median per 24 h 43.6/2.25. Trap catch was highly heterogeneous, and the majority of *M. sorbens* were caught during two trap ‘events’ (3- or 4-day periods). A small number of studies report *M. sorbens* trapping. Human faeces as an attractant in the Bristow trap caught totals of 250, 1138 and 152 *M. sorbens* over three studies, giving 16.7/11, 71.1/66 and 6.3/6 (mean/median) per 24 h^17^. In two studies that tested blue and yellow sticky traps, as well as sticky pots (equivalent to Bristow traps), blue sticky traps caught a mean of 4.87 and then 4.92 *M. sorbens* per trap night (24-h period)^12^. Both studies report these as fly monitoring traps, although use of blue sticky cards for control is suggested by the latter study. Fly populations including *M. sorbens* were monitored in The Gambia using fish-baited traps, and the median (range) of the adjusted geometric mean numbers of *M. sorbens* per trap per day was 9.80 (2.29–37.3) versus 5.98 (2.41–38.3), in the wet and dry seasons respectively^2^. A study in Ethiopia followed the methods of Emerson^2^, reporting catches of *M. domestica* and *M. sorbens* of just 15.5% and 9.1%, respectively, of the total catch from beef-baited traps^20^. The mean *M. sorbens* catch can be calculated as 28.8 per trap per night. Statistics reported by trapping studies are variable, which hampers comparisons between studies, and clearly trap catch is dependent on study site. Overall, however, the Bickford Bucket mean/median catch rates reported here are broadly comparable with other published studies.

Like most calyptrate dipterans, *M. sorbens* has a short generation time and populations can increase rapidly. It has been estimated that for such species, to reduce the population by 50–90% theoretically requires that 24–58% of the fly population be removed by trapping each day^26^. Therefore, it is more important to understand what proportion of the local *M. sorbens* population is being caught than the absolute catch rate. This is best achieved using mark-release-recapture studies^1,27^. Characteristics of the trap catch should also be examined, specifically the reproductive age of caught females should be measured^28^ to determine if they have oviposited. If the attractant lures females before they have oviposited (for example if the lure represents a food or oviposition source) it is more likely to suppress the population. The local proportion of flies that are carrying *C. trachomatis*, and their propensity to be trapped, will also influence whether trapping can control disease. If *C. trachomatis* carriage is biased towards female flies for instance, which represent a higher proportion of the trap catch, the impact of trapping on disease control may be amplified. To definitively determine if mass trapping a disease vector can control disease, trapping studies with epidemiological outcomes are warranted (i.e. measuring changes in the prevalence of trachoma following implementation of a trapping program). All of these strategies were beyond the scope of these studies but should be considered in future trials using this trap.

Trapping studies are renowned for heterogenous data. Here, no measured variable accounted for the variation in *M. sorbens* catch, including the two trapping events in which 1399 and 1881 *M. sorbens* were caught over 3- and 4-day periods. Relative humidity, ambient temperature, light intensity and the presence of wind/rain were all measured at trap collection and, therefore, do not necessarily reflect conditions throughout trap deployment; continual data loggers should ideally have been used. For this reason, we cannot discount the possibility that these environmental factors did affect fly catch. When constructing the Bickford Bucket, only approximate measurements were used when placing and cutting holes in the bucket. It is possible that the resulting slight variation in trap dimensions affected trap catch. Other, unmeasured, variables could have played a role—*M. sorbens* are highly attracted to people, so the number of people present or around the house during trapping could influence fly catch, especially children, to whom *M. sorbens* are particularly attracted. Further, we cannot exclude that another potent attractant, human faeces, was present locally and influenced fly behaviour.

When the performance of the Bickford Bucket/Buzz lure trap in terms of *M. sorbens* catch is evaluated in the wider context of vector control tool, it merits further investigation. The trap components were locally available and would be so in most study settings (empty water bottles, a bucket and tape), as well as being cheap (approximate total cost 90 Ethiopian birr, £ 2.50). It is anticipated the trap would be durable with minimal maintenance, very unlikely to saturate (the 4-week lure lifespan would expire before the 5-L ‘trap collector’ is full) and easy to empty. The trap catch is well contained and therefore unlikely to pose a hygiene risk, while overall the trap (and lure) is safe and harmless for use outside the house and easy to maintain by householders. The Buzz lure itself retails online at around £ 2.40 for one sachet, which lasts 4 weeks. Although it is probably unavailable in many trachoma-endemic countries, shipping is relatively straightforward as sachets are lightweight (4 g each) and prior to solubilisation, sachets have a long shelf life (2 years when stored in a moisture-free environment, or in an unopened sachet). Over the course of our studies, participants were found to engage well with the idea of trapping, taking care of and pride in their trap. Arthropods other than *M. sorbens* were not identified due to time constraints, however, The Buzz lure is a generic ‘fly’ attractant (meaning filth flies, calyptrate dipterans). Other probable filth flies were observed in the fly catch; therefore, the trap may increase general household hygiene and possibly reduce mechanical transmission of fly-borne pathogens other than trachoma.

It is possible that trap catch could be increased by relatively minor adjustments, for example changing the colour of the bucket, specifics of the trapsite, or the height of the trap. Blue was used here because blue buckets were easily available locally, and in some insect species, blue with the specific spectral reflectance at 466 nm is attractive. Although there have been contradictory studies, in one study considerably more house flies were collected in blue than in white or black traps^29^, another found white and blue attractive to *M. domestica* in light tunnel assays^30^, and recently blue sticky pot traps (Bristow traps) were found to catch more *M. sorbens* than yellow versions of the same^12^. Blue buckets have been used to build fish-baited attractant fly traps^2^. Use of specific shades of blue with particular spectral reflectance may optimise catch. We placed traps on the ground near the house, in a sheltered/protected location that was not an inconvenience to the householders. Close proximity to the house was favoured because of the flies’ strong attraction to people. The Bickford Bucket would need to be slightly modified for stability before changing trap height. While *M. sorbens* seek oviposition sites at ground level, they presumably seek food sources at varying heights including human eye-level. A study in The Gambia found no influence of trap height (floor versus hanging at 1–1.5 m) on the number of Muscid flies caught their fish-baited exit trap^2^, while trap catches of the closely related *M. vetustissima* were found to be inversely related to height^31^.

Although mass trapping is not yet commonly deployed for vectors of disease, in some instances it has been very successful^32–34^, although failures have been documented^35^. The success of trapping the vector of African trypanosomiasis, tsetse flies, is partially attributed to the ‘K-type’ life history of this species, a slow reproductive rate that is unusual in insects. In the context of agricultural/horticultural pest control, it is known that successful trapping is dependent on several factors, including: the density and efficiency of traps as well as lure strength that must be sufficient to catch many insects, the lures must be able to outcompete natural sources of attraction, traps should catch insects before they mate or oviposit, the trap and lure should remain effective throughout the adult insect’s lifespan (emergence to mating), and the costs of trapping should be less than the benefits of alternative ways of increasing crop yield^21^. Further, trapping is known to be most effective when pest density is low, indeed trapping high density pests has been met with failure^36,37^, and in some instances it is useful to reduce by insecticide prior to trapping isolated populations. While it is important to consider these criteria, the Bickford Bucket was designed to produce a fly control tool for *M. sorbens* quickly, conveniently and cheaply. Determining the optimal trap density becomes less important if the trap is cheap, easy to use and people are willing to have it placed outside their house and maintain it. Future studies could explore replacing The Buzz lure with *M. sorbens*-specific semiochemicals^17^, although developing such a lure to a field-ready state will require significant research investment, with the financial and timescale implications.

Finally, few tools exist for the control of *M. sorbens*. Deploying the Bickford Bucket alongside other control measures, for example, after insecticide spraying, or alongside implementation of WASH strategies or a fly repellent technology^38^, may allow better fly control. We are initiating such a study in the Stronger-SAFE Phase 3 cluster-randomized trial of double-dose oral azithromycin combined with targeted transmission-interrupting strategies (behaviour change to change facial hygiene practices and entomological control including traps and insecticide-treated headwear).

## Methods

### Pilot laboratory study

**Figure 4:**
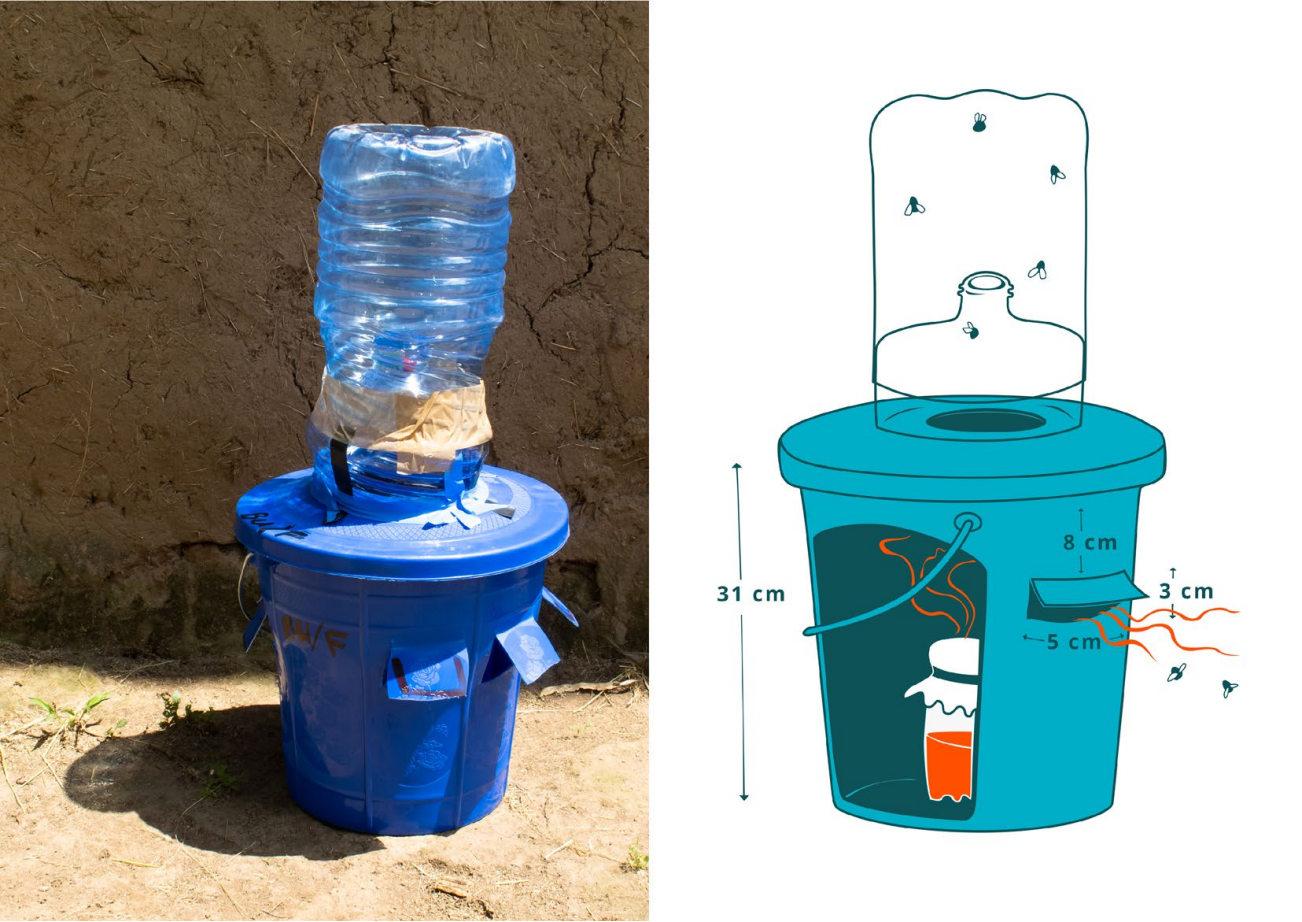
Bickford Bucket (BB) trap, a homemade trap designed from a 5-L water bottle and a bucket. Inside the bucket, a lure is presented in another (250 mL) water bottle, covered with mesh. Image courtesy of Iain Robinson (Iain-robinson.com).

Six traps were chosen for initial efficacy testing in laboratory studies (Fig. 1). Four were commercially available and bought online: The Buzz (STV International Ltd; Thetford, UK), Fly Terminator Pro (Starbar Products, Schaumburg, Illinois), Red Top Fly Catcher (Ashmoat Ltd, Athelington, UK), insect bait trap with nylon screen, BugDorm (no bait included) (MegaView Science Co., Ltd., Talchung, Taiwan). Baited commercial traps used dry, food-based formulations (The Buzz, a mixture of non-toxic food proteins and carbohydrates; Fly Terminator Pro, a mixture including Z-9-tricosene, Egg, trimethylamine, indole and sucrose; Red Top, protein-based) to which water is added and the formula aged before use. The Buzz lure is also known as STV Fly Lure. A further two were homemade trap designs: a ‘field expedient’ bottle trap (‘plastic water bottle fly trap [multihole model]’^39^), and a custom-designed trap, the Bickford Bucket (BB) trap. The BB trap was composed of a 10-L blue bucket, 5-L and 600-mL water bottles, and was inspired by the design of an odour-baited sticky trap for *Anopholes arabiensi*s^40^ and a small-scale fly trapping study in Kenya^41^. To assemble the BB, holes (approximately 5 × 3 cm; six holes) were cut into the bucket such that they were just above the height of the lure-containing (600 mL) bottle inside. The top was cut off the 5-L bottle and this section was firmly attached with electrical tape over a hole in the bucket lid (Fig. 4, full assembly instructions Supplementary Fig. S1). The rest of the bottle could then be loosely taped on top and removed to assess fly catch; tape could be easily replaced or bolstered with extra, but this was generally not necessary.

Each trap was tested for one period of 24 h in a BugDorm-6 Insect Rearing Cage (60 × 60 × 180 cm, BugDorm [MegaView Science Co., Ltd., Talchung, Taiwan]); traps were tested individually and serially over 6 days. Traps were assembled inside the cage, then 108 laboratory-reared, mixed-sex *M. sorbens* were released into the cage and at the opposite end to the trap. Commercial traps were prepared according to manufacturer’s instructions. Traps with no bait (insect bait trap with nylon screen, field expedient bottle trap and BB trap) were baited with 50 g (Ascher Portable Digital Scale) faeces, the breeding medium of *M. sorbens*, from one donor. Faeces samples were placed into clear plastic cups covered by netting (Nylon dress net, John Lewis, UK) to prevent fly contact.

*Musca sorbens* (narrow frons type) were used from the colony at the London School of Hygiene and Medicine (rearing procedures in “Supplementary Methods”). Flies were acclimatised to the test room for 12 h prior to testing; flies per trapping event were age standardized. Traps were assessed for field testing based on convenience of use, total fly catch, and how appropriate we considered them to the field setting. For this, we evaluated aspects including how easily the trap would saturate/how large the fly collection chamber was, how stable the trap was, whether it was flimsy, etc.

### Field studies

#### Study location and ethics

Field testing took place in a rural kebele (village) near Shashemene (Oromia, central Ethiopia; 7° 17′ 54.7″ N, 38° 35′ 07.9″ E). The study was approved by the LSHTM ethics committee (reference number 14688) and the Oromia Regional Health Bureau Ethics Committee. Written informed consent was provided by household heads for the household to participate, all research was performed in accordance with relevant guidelines and regulations. Field study 1 took place between 05/07/2018 and 22/08/2018 (rainy season); field study 2 between 23/09/2018 and 12/11/2018 (some rains); and field study 3 between 14/01/2019 and 09/03/2019 (hot/dry season). Ambient temperature (°C), relative humidity (%), light intensity (lux), and presence/absence of wind/rain were recorded at each trap setting/collecting event.

#### Field study 1

Of the commercial trap designs tested in the pilot laboratory study, The Buzz (herein referred to as The Buzz/L [commercial lure]) and the Fly Terminator Pro (herein referred to as the Terminator) were selected for field testing. In addition, The Buzz and the BB trap were tested with a faeces bait (herein referred to as The Buzz/F [faeces bait] and the BB/F [faeces bait]). The Bristow trap, another homemade design^17^, was added to this selection, as a positive (faeces bait) and negative (non-baited) control (Fig. 3). The Buzz commercial lure was prepared 24 h in advance and used, per trap, for 4 weeks before replacing. Faeces baits (50 g, Ascher Portable Digital Scale) were from one donor and replaced at each deployment. Six houses, all at least 500 m apart, were selected as trap sites. Traps were rotated between trap sites in a Latin Square design, five times (*n* = 30 trap events), each testing period being 24 h. Trap setting and collecting was done in the morning; traps were placed beneath an upturned waste-paper basket (Bristow trap/The Buzz), or surrounded by branches, to protect them from rain and interference from e.g. domestic livestock (Terminator/BB). At trap collection, trap catch was knocked down with acetone for transit back to the laboratory, where total male/female *M. sorbens* and total other arthropods were counted.

#### Field study 2

In field study 2, one commercial and one homemade trap design was tested; The Buzz was tested with it’s own lure (Buzz/L) and with water (Buzz/N), while the BB was tested with a faeces bait (BB/F), with water (BB/N), and with The Buzz lure (BB/L) (Fig. 3). Here, lures were presented in a 500 mL water bottle inside the bucket, with the top cut off and netting (Nylon dress net, John Lewis, UK) used to protect the contents (Supplementary Fig. S1). The Buzz commercial lure was prepared 24 h in advance and used, per trap, for 4 weeks before replacing. Traps baited with only water were negative controls for commercial water-based lures, faeces baits (50 g, Ascher Portable Digital Scale) were from three consistent donors and replaced at each deployment. These five trap combinations were tested in the same geographical area but at ten new households at least 500 m apart. Traps were rotated between trap sites in a Latin Square design, with two Latin Squares running simultaneously across the ten trap sites. A total of six Latin Squares were completed (*n* = 30 trap events), traps were set and collected on Mondays and Thursdays so testing periods were 4 and 3 days. BB traps were surrounded by branches to protect them from interference, The Buzz traps were protected by upturned waste-paper baskets. At trap collection, trap catch was knocked down with acetone for transit back to the laboratory, where total male/female *M. sorbens* and total other arthropods were counted.

#### Field study 3

In field study 3, two homemade trap designs were tested: the BB trap was again tested with a faeces bait (BB/F), with water (BB/N), and with The Buzz lure (BB/L), while the Bristow trap was deployed as a positive (faeces bait) and negative (non-baited) control (Fig. 3). The Buzz commercial lure was prepared 24 h in advance and used, per trap, for 4 weeks before replacing. Traps baited with only water were negative controls for commercial water-based lures, faeces baits were as for field study 2. The Bristow traps were protected by upturned waste-paper baskets, the BB by branches. Study design was as with field study 2, the study was conducted in the same geographical area but at ten new households at least 500 m apart.

### Statistical analysis

Data were entered into Open Data Kit (ODK) using tablets (Samsung Galaxy Tab E) before being exported and analysed in Stata (v. 15, StatCorp). Trap catch data were discarded if the trap had been disturbed during deployment, trap catch from field studies 2 and 3 was normalised to represent catch per 24 h. Negative binomial regression was used to model the association between trap and the number of flies caught, this model accounted for the over-dispersed nature of trap catch data. Household (trapsite) was treated as a random effect for all models, except in testing the association between ‘other arthropod’ trap catch, trap type and the presence of sheep in the compound in field study 3, where household was treated as a fixed effect. Likelihood ratio tests were used to test associations between trap catch and measured variables; each variable was added separately. Other than trap type, variables tested were: presence of human/animal faeces in compound, distance of faeces if ‘yes’, presence of latrine at trapsite, presence and type of any new waste in compound, presence and type of any livestock in compound, presence of wind/rain (at trap collection only), history of rain during trap deployment, ambient air humidity/temperature (at trap collection only). ﻿The effect of trap bait on the likelihood that a trapped *M. sorbens* was female was analysed using logistic regression.

## Supplementary Information

Supplementary Information.

Supplementary Figure S1.

Supplementary Figure S3.

## Data Availability

The datasets generated during the current study are available from the corresponding author on reasonable request.
