# Supplementary material for: Towards an odour-baited trap to control Musca sorbens, the putative vector of trachoma
Source: Sci Rep. 2021 Jul 9;11:14209. doi: 10.1038/s41598-021-91609-1 (PMC8271020; doi:10.1038/s41598-021-91609-1)
Supplement: Supplementary file 1 — Supplementary Information. [file 41598_2021_91609_MOESM1_ESM.docx]

# Towards an odour-baited trap to control *Musca sorbens*, the putative vector of trachoma

*Ailie Robinson

Affiliation for study: Department of Disease Control, London School of Hygiene and Tropical Medicine, Keppel Street, London, WC1E 7HT, UK.

[Ailie.robinson@lshtm.ac.uk](mailto:Ailie.robinson@lshtm.ac.uk)

Jack Bickford-Smith

Affiliation for study: Department of Disease Control, London School of Hygiene and Tropical Medicine, Keppel Street, London, WC1E 7HT, UK.

[Jack.Bickford-Smith@lshtm.ac.uk](mailto:Jack.Bickford-Smith@lshtm.ac.uk)

Oumer Abdurahman Shafi

Affiliation for study: The Fred Hollows Foundation, P.O. Box 6307, Addis Ababa, Ethiopia. International Centre for Eye Health, Department of Clinical Research, London School of Hygiene and Tropical Medicine, Keppel Street, London, WC1E 7HT, UK.

[Oumer.Abdurahman@lshtm.ac.uk](mailto:Oumer.Abdurahman@lshtm.ac.uk)

Muluadam Abraham Aga

Affiliation for study: The Fred Hollows Foundation, P.O. Box 6307, Addis Ababa, Ethiopia.

[maga@hollows.org](mailto:maga@hollows.org)

Gemeda Shuka

Affiliation for study: The Fred Hollows Foundation, P.O. Box 6307, Addis Ababa, Ethiopia.

[gshuka@hollows.org](mailto:gshuka@hollows.org)

Dereje Debela

Affiliation for study: The Fred Hollows Foundation, P.O. Box 6307, Addis Ababa, Ethiopia.

Current affiliation: Population Service International, Addis Ababa, Ethiopia.

[derejetola1@gmail.com](mailto:derejetola1@gmail.com)

Gebreyes Hordofa

Affiliation for study: The Fred Hollows Foundation, P.O. Box 6307, Addis Ababa, Ethiopia.

Current affiliation: Netherlands Development Organization

[geberayeslemma@gmail.com](mailto:geberayeslemma@gmail.com)

Wondu Alemayehu

Affiliation for study: The Fred Hollows Foundation, P.O. Box 6307, Addis Ababa, Ethiopia.

[walemayehu@hollows.org](mailto:walemayehu@hollows.org)

Virginia Sarah

Affiliation for study: Global Partnerships Executive, The Fred Hollows Foundation, 12-15 Crawford Mews, York Street, London W1H1LX

vsarah@hollows.org

Anna Last

Affiliation for study: Department of Clinical Research, London School of Hygiene and Tropical Medicine, Keppel Street, London, WC1E 7HT, UK.

[Anna.Last@lshtm.ac.uk](mailto:Anna.Last@lshtm.ac.uk)

David MacLeod

Affiliation for study: Department of Infectious Disease Epidemiology, London School of Hygiene and Tropical Medicine, Keppel Street, London, WC1E 7HT, UK.

[david.macleod@lshtm.ac.uk](mailto:david.macleod@lshtm.ac.uk)

Matthew J. Burton

Affiliation for study: International Centre for Eye Health, Department of Clinical Research, London School of Hygiene and Tropical Medicine, Keppel Street, London, WC1E 7HT, UK.

[matthew.burton@lshtm.ac.uk](mailto:matthew.burton@lshtm.ac.uk)

James G. Logan

Affiliation for study: Department of Disease Control, London School of Hygiene and Tropical Medicine, Keppel Street, London, WC1E 7HT, UK.

[James.Logan@lshtm.ac.uk](mailto:James.Logan@lshtm.ac.uk)

*Correspondence to Ailie Robinson

### Supplementary Methods: *Musca sorbens* colony maintenance

The colony of *Musca sorbens* ﻿Weidmann (Diptera: Muscidae) used originated from the field site of this study, in the kebeles of Shashemene Woreda, Oromia, Ethiopia. It has been kept in the insectaries at the London School of Hygiene and Tropical Medicine since importation of pupae on 26/04/2018 (Common Veterinary Entry Document reference number CVEDA.GB.20180003201-V1). Adult flies were fed on a regular supply of organic whole milk powder (Buy Whole Foods Online Ltd, Ramsgate, UK) and granulated sugar (Tate and Lyle, London, UK), each presented in a glass petri dish (80 x 15 mm soda lime glass, DURAN®, DWK Life Sciences [Kimble], Mainz, Germany). Dry food was intermittently supplemented with long-life ultra-high temperature processing (UHT) whole milk (Co-op Food, Manchester, UK), soaked into cotton wool (Scientific Laboratory Supplies, Nottingham, UK) and presented in a 60-ml Sterilin® pot (Sterilin® Ltd, Newport, UK). Adult flies were kept in 60-cm cubic gauze-covered cages (Bugdorm®, Megaview, Taiwan) in a climate-controlled room (26±1°C, 40±10% RH, LD 12:12) and had access to distilled water (purified using a Milli-QSP system; ﻿Millipore, Milford, MA, U.S.A.). Water was presented in closed 500 mL bottles (emptied Highland Spring Sparkling Mineral Water bottle, Perthshire, UK) with very small holes pierced in the lid, propped upside-down on top of cotton wool in a glass petri dish. This system allowed slow and controlled leakage of water into the cotton wool over several days. Juvenile stages were reared using human faeces. Human faeces were presented to adult flies inside a 12-oz compostable soup container (Vegware, Edinburgh, UK) for 24-48 hours, loosely covered by an identical, but upside-down, pot with a hole cut in it to allow fly access. After the oviposition period, stool was thoroughly covered with sand (Children’s Play Sand, Argos, Milton Keynes, UK), removed from the breeding cage and placed into an ‘emerging jar’: a 5-litre plastic water bottle (Fairbourne Springs Mineral Water, Co-op Food, Manchester, UK) with the top cut off. Sufficient sand was added to completely fill the compostable pot and overflow some sand into the emerging jar. This ensured that any larvae migrating away from the faeces would still pupate. The emerging jar was covered using cut-off tights (Women's Everyday Nude Silk Sheer Soft 20 Denier Tights, MANZI, Amazon UK, London, UK). After pupae appeared in the sand, water (milipore filtered, soaked into cotton wool in a Sterilin® pot) and granulated sugar (offered in an upside-down Sterilin® pot lid) were added into the emerging jar. Emerging adults were collected from the emerging jar using an electronic pooter and placed into 30-cm cubic gauze-covered cages (Bugdorm®, Megaview, Taiwan) until use, with access to sugar, powdered milk and water.

### Supplementary Figure S1: construction of the Bickford Bucket

This homemade trap is composed of a 10-litre blue bucket, 5-litre empty water bottle, small water bottle (500/600 ml) for the lure inside, and tape to hold everything together. Electrical tape is preferable if available. The holes in the side of the bucket are cut such that the cut section can be bent up to function as a baffle, which prevents flies from using light to find these openings to escape the bucket, and instead fly upwards into the large bottle.

### Supplementary Table S2: *Musca sorbens* caught in field study 3

Summary statistics for *M. sorbens* caught per trap type, n=trap events (three- or four-day deployment)

| Trap | n | Total female | Total male | Total^A^ | Mean^B^ (SD)/24-hours | Median^B^ /24-hours (IQR) | Mean^B^/7 days | Median^B^ /7 days (IQR) |
| --- | --- | --- | --- | --- | --- | --- | --- | --- |
| Bristow/N | 29 | 13 | 10 | 23 | 0.23 (±0.83) | 0 (0-0) | 1.61 | 0 |
| Bristow/F | 27 | 388 | 152 | 562 | 6.40 (±9.99) | 2.50 (0.33-7.75) | 44.8 | 17.5 |
| BB/N | 20 | 0 | 1 | 1 | 0.02 (±0.07) | 0 (0-0) | 14 | 0 |
| BB/F | 24 | 52 | 19 | 76 | 0.95 (±1.76) | 0 (0-0.71) | 6.65 | 0 |
| BB/L | 26 | 2645 | 1193 | 3848 | 43.64 (±137.10) | 2.25 (0.25-12.67) | 305.48 | 15.75 |
| Total | 126 | 3098 | 1375 | 4510 | 10.61 (±63.82) | 0 (0-2.50) | 74.27 | 0 |

^A^Total *M. sorbens* includes flies of unknown sex.

^B^Total *M. sorbens*; 3- or 4- day trap catches have been adjusted to represent per 24-hour catches.

### Supplementary Figure S3: Total *M. sorbens* catch per trap event

Two trap events (indicated here by 1 and 2) occurred per collection date, per trap type, as two Latin Squares ran simultaneously across the ten trapsites. Trap abbreviations are: Bristow/N=Bristow trap/no bait, Bristow/F= Bristow trap/faeces bait, BB/N=Bickford bucket/no lure (water), BB/F=Bickford bucket/faeces bait, BB/L=Bickford bucket/lure (Buzz). Trap events (three- or four-day deployment) per trap type given as data labels in (A); those are equal for A/B.
