# Supplementary figures and images for: Towards an odour-baited trap to control Musca sorbens, the putative vector of trachoma
Source: Sci Rep. 2021 Jul 9;11:14209. doi: 10.1038/s41598-021-91609-1 (PMC8271020; doi:10.1038/s41598-021-91609-1)

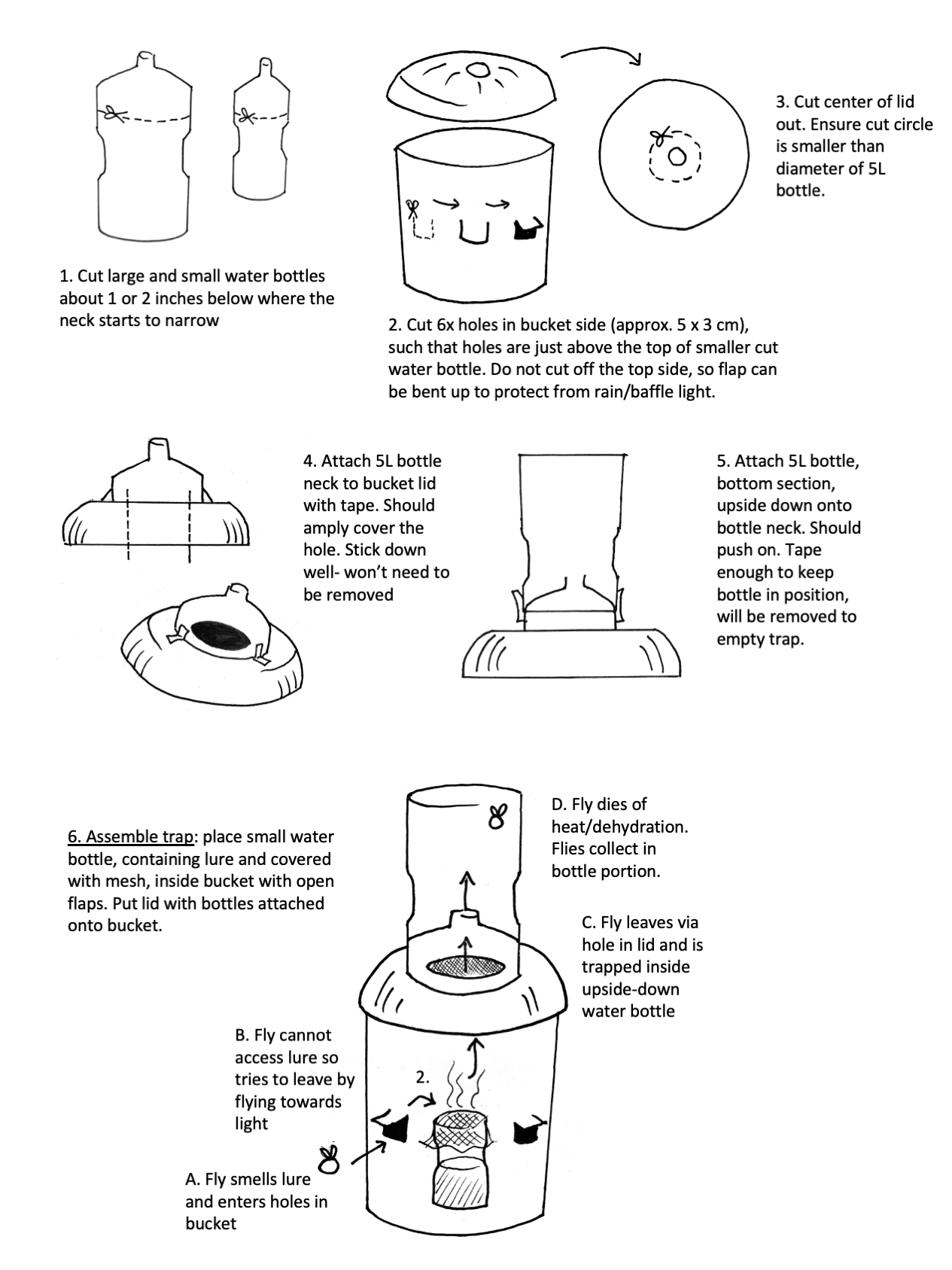

Supplement: Supplementary file 2 — Supplementary Figure S1. [file 41598_2021_91609_MOESM2_ESM.tiff]

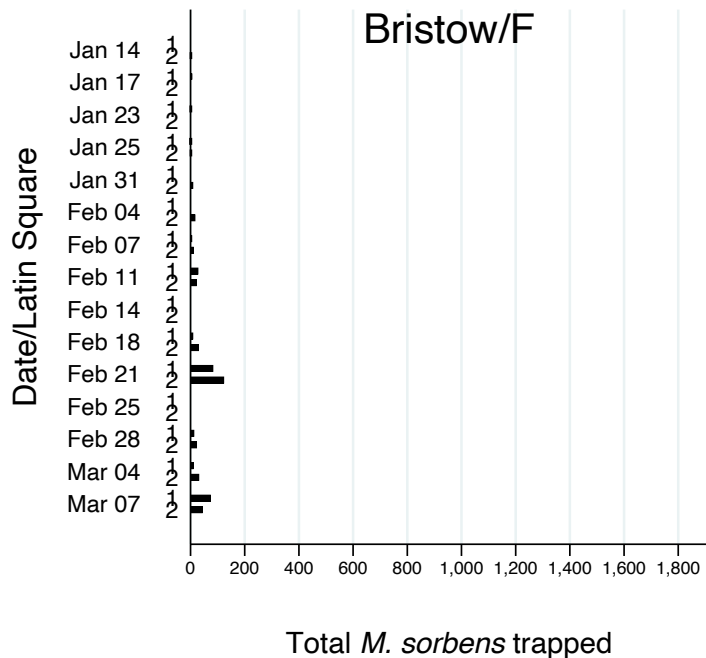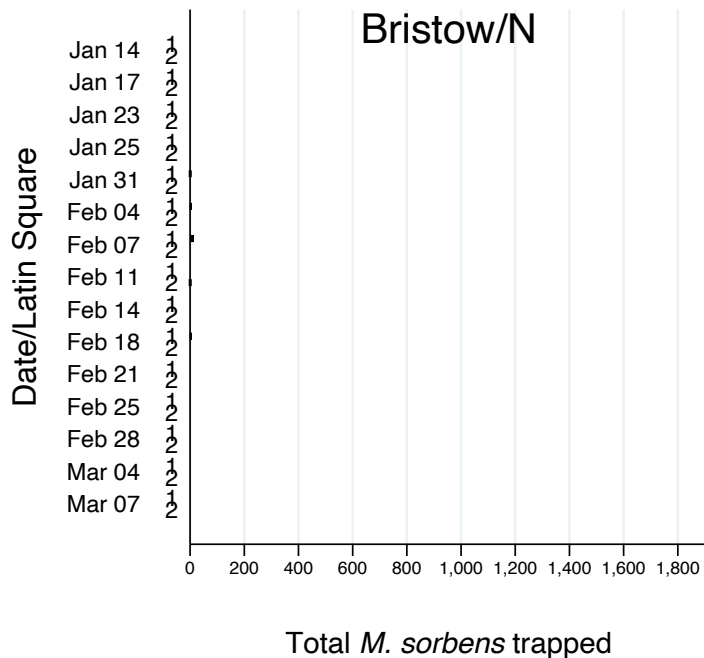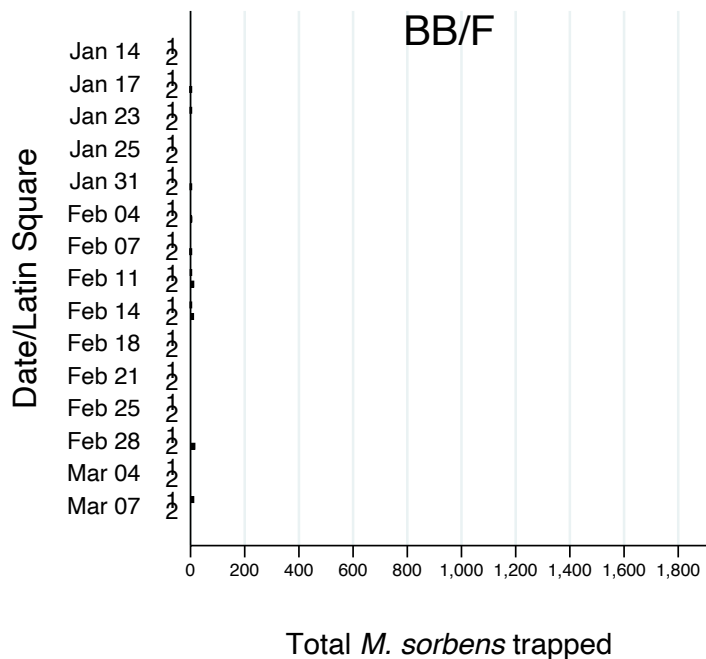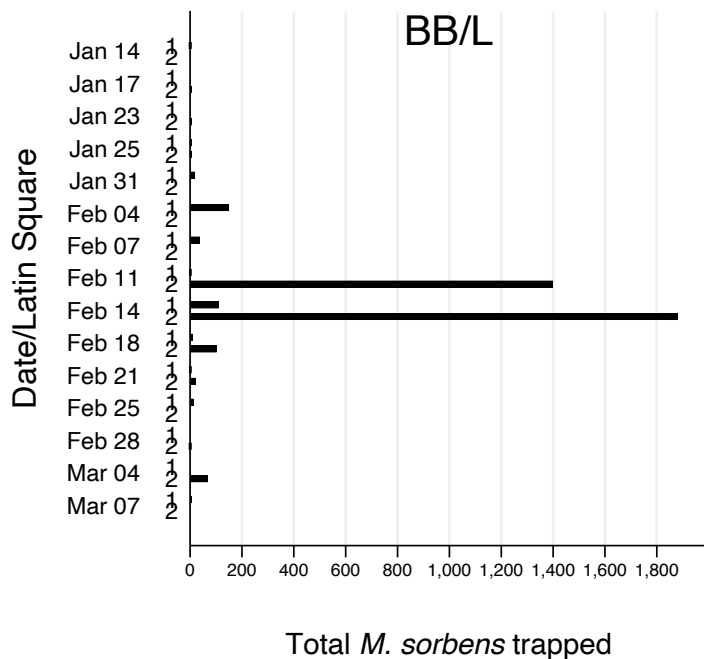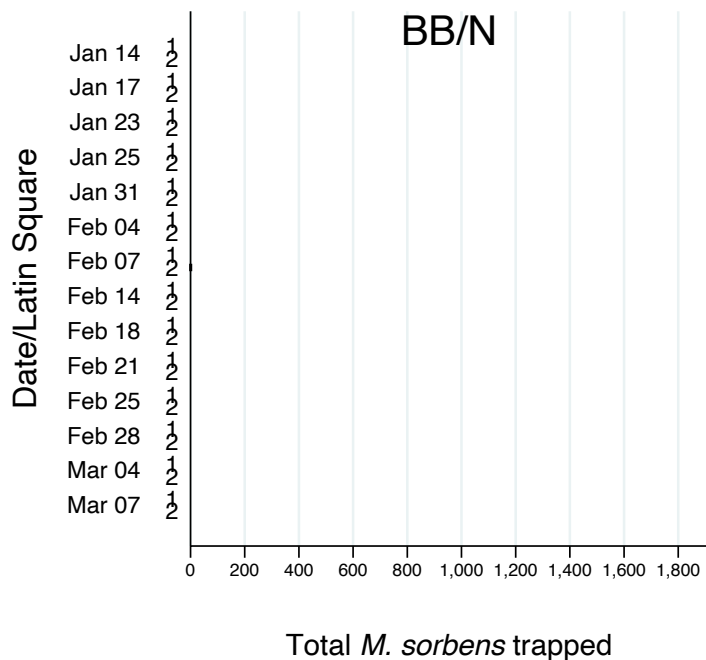

Supplement: Supplementary file 3 — Supplementary Figure S3. [file 41598_2021_91609_MOESM3_ESM.pdf]
